# Nutritional characterization of carobs and traditional carob products

**DOI:** 10.1002/fsn3.776

**Published:** 2018-10-04

**Authors:** Eleni Papaefstathiou, Agapios Agapiou, Stelios Giannopoulos, Rebecca Kokkinofta

**Affiliations:** ^1^ Department of Chemistry University of Cyprus Nicosia Cyprus; ^2^ State General Laboratory Nicosia Cyprus

**Keywords:** carob products, *Ceratonia siliqua L*., chemical composition, chemometrics, elemental profiling, functional food

## Abstract

Twenty traditional carob products were measured for their nutritional composition, and their results were compared with the pulp of Cypriot carob cultivars. Moisture, ash, fat, proteins, sugars, dietary fibers, minerals, caffeine‐theobromine, carbohydrates, and energy value were determined. Fluctuations of the nutritional composition values based on the ingredients’ chemical synthesis and product manufacturing process were noted. Only 60% of the products had a label indicating their nutritional value, and the majority of them (75%) were consistent with that of labeling. Chemometric analyses distinguished the carob products according to their type and the discriminator components highlighted their particular nutritional value. Carobs can be characterized as functional foods with low‐fat content, high content in dietary fibers, and high content and/or source of minerals; however, carob products partially satisfied those health and nutritional claims as expected. This pilot research contributes to the nutritional estimation of carob and highlights the traditional carob products.

## 1 INTRODUCTION

The scientific name of carob tree, *Ceratonia siliqua L.,* derives from the Greek word “*Kera,*” which relates to the keratomorphic shape of the fruit, and the Latin word *siliqua*, which refers to the pods’ hardness and shape. Carob tree is cultivated in most Mediterranean countries, mainly in mild and dry areas. World production of carob is estimated at 160,000 tons per year (Goulas, Stylos, Chatziathanasiadou, Mavromoustakos, & Tzakos, 2016). Spain produces the largest quantities, followed by Italy, Portugal, Morocco, Turkey, Greece, Cyprus, and Lebanon (Food and Agriculture Organization of the United Nations).

Carob fruits are characterized by high sugar content (48%–56%) (mainly sucrose, glucose, and fructose), 3%–4% protein, a low‐fat content (0.2%–0.6%) (Batlle & Tous, 1997), low content of alkaloids, and high content of dietary fibers, especially in the seeds (Ortega et al., 2009). Specifically, the pulp is composed of sugars, polyphenols (e.g., tannins, flavonoids, phenolic acids), and minerals (e.g. K, Ca, Mg, Na, Cu, Fe, Mn, Zn), whereas the seed contains proteins, dietary fibers, polyphenols, and minerals and is free of gluten.

Carob powder is a valuable source of vitamins E, D, C, Niacin, B6, and folic acid; vitamins A, B2, and B12 are provided in lower levels. Carob powder oil is composed of 17 fatty acids, mainly oleic, linoleic, palmitic, and stearic acid at 40.45%, 23.19%, 11.01%, and 3.08%, respectively (Youssef, El‐Manfaloty, & Ali, 2013). A number of cyclitols are also present in carob beans. The major cyclitol is D‐pinitol (3‐O‐methyl‐D‐chiro‐inositol) with multiple health benefits (Goulas et al., 2016).

The endosperm of the seed contains the water‐soluble mucus, known as locust bean gum (LBG), which is a polysaccharide (galactomannan) consisting of 16%–20% D‐galactose and 80%–84% D‐mannose. It is created from seed processing and is a natural additive (E410) (Salinas, Carbas, Brites, & Puppo, 2015). It is widely used in the food industry as thickener, stabilizer (Lazaridou, Biliaderis, & Izydorczyk, 2001), and gelling or dispersing agent, and its labeling is compulsory. LBG has many applications in cosmetics, pharmaceuticals, film emulsions, paints, polishes, ceramics, and adhesives (Batlle & Tous, 1997).

Many studies have shown that carobs and their products can promote human health and help prevent specific chronic diseases. In particular, they show antiproliferative and apoptotic activity against cancer cells, they are suggested to treat diarrhea symptoms, and they present antihyperlipidemia and antidiabetic effects due to their high antioxidants, polyphenols, and high content in fibers (Theophilou, Neophytou, & Constantinou, 2017). Therefore, they are considered ideal food for people with diabetes (Youssef et al., 2013). Carob flour (from carob seeds) is used to manufacture dietetic products and products for celiac patients (gluten‐free products) (Tsatsaragkou & Gounaropoulos, 2014).

Carob is an indigenous drought‐ and temperature‐tolerant tree cultivated in Cyprus for centuries. In the past, it significantly benefited the agricultural economy of the island. It was widely known as the “black gold” of Cyprus. In recent years, carob's health benefits and nutritional value are being highlighted and therefore traditional carob‐based food products end up in the market. In Cyprus, many traditional carob products are produced (Table 1); the most widely known is the carob syrup (charoupomelo) which is exported to many countries. The literature work on Cyprus carob cultivars is very limited. Currently, the geographical origin and type of *Ceratonia siliqua L*. material (flesh and pods), from Cyprus and other countries, were discriminated based on Fourier transform infrared (FTIR) spectroscopy and chemometrics (Christou, Agapiou, & Kokkinofta, 2018).

**Table 1:**
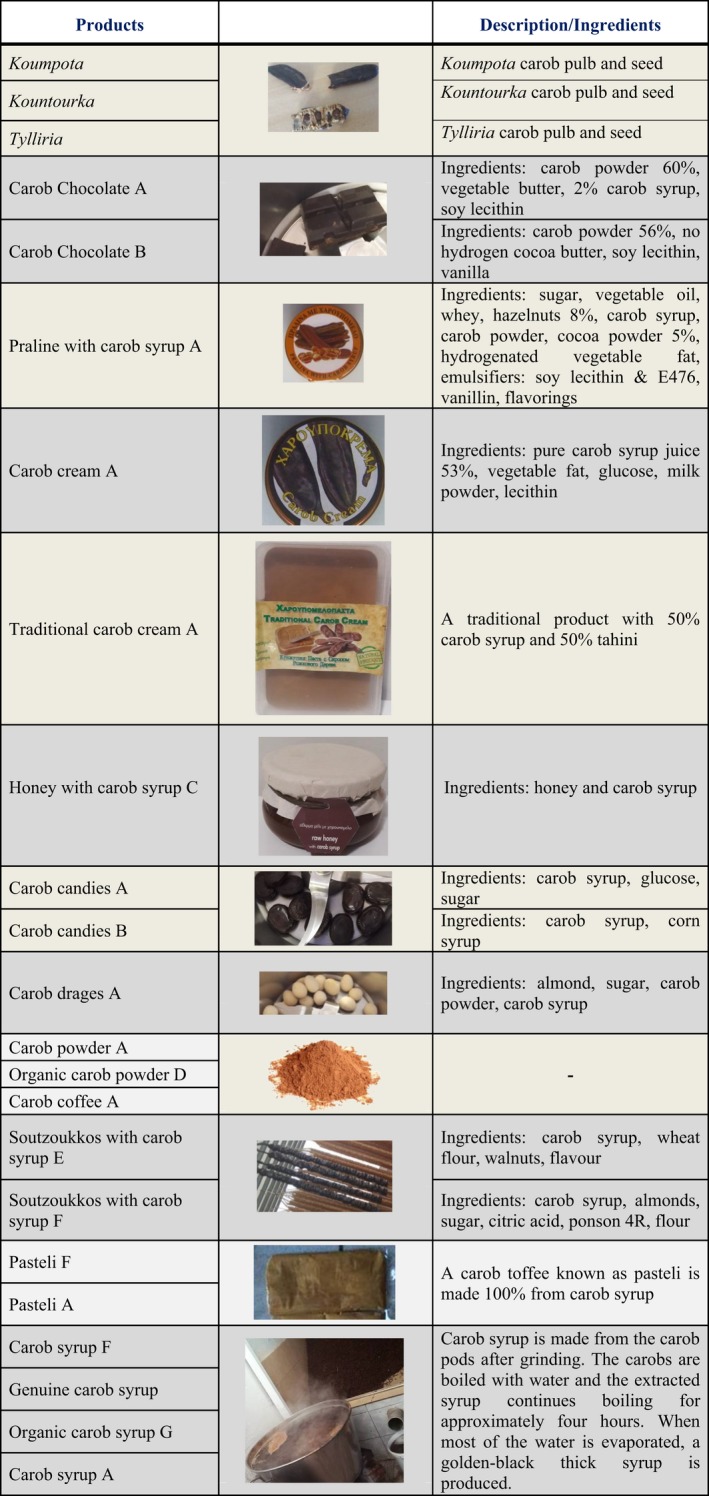
The products that were analyzed for their micro‐ and macronutrients

According to the modern market needs and progressions, the next years, carobs’ importance is expected to enhance globally due to: the cocoa shortage (Loullis & Pinakoulaki, 2018), the trend toward health and nutrient supplements, the need for biological and gluten‐free products, along with the necessity for natural hydrocolloids.

To our knowledge, little is known about standardization of traditional carob products. This is due to nonindustrial food processing methods, limited manufacturing practices, and lack of long‐term documented research. Highlighting the importance of traditional products is a modern food industry trend (protected designation of origin, PDO and protected geographical indication, PGI), along with that of discovering or creating new functional foods (Regulation (EU) No 1151/2012; Luykx & van Ruth, 2008). Therefore, the aim of the present study is the adequate study of the nutritional value of carobs and carob products with origin from Cyprus using official and approved analytical methods and chemometrics. Toward this, the issue of insufficient labeling of traditional carob products is highlighted and data for evaluating their efficacy as functional foods are provided. The examined products are presented in detail in Table 1.

## 2 MATERIALS AND METHODS

All the analyses were performed in duplicate or triplicate, under the same laboratory conditions, in an accredited laboratory using either official or accredited/validated methods. Moisture, ash, fat, proteins, sugars, dietary fibers, minerals, and caffeine‐theobromine were determined in all samples, and the results were compared to that of domestic carob fruits, widely found in the island of Cyprus (cultivars: *Koumpota, Kountourka, and Tylliria*). Carbohydrates were calculated by difference, and energy value was calculated with the energy calculation factors.

### 2.1 Experimental procedures

Moisture was determined by thermogravimetric analysis (ISO 1422:1997, AOAC 925.10 and AACC Method 44‐15A, AOAC 920.151). Gallenkamp oven was calibrated at 103°C and 130°C, and Jeio Tech vacuum oven was used.

Ash was determined by Dry Oxidation—Incineration process according to method AOAC 14‐098 and (Kirk & Sawyer, 1991). Carbolite furnace was employed at 550°C along with infrared lamp, bunsen burner, and porcelain beakers.

Fat was determined with the Soxhlet method (Büchi extraction Unit E‐816 SOX), based on AOAC Official Methods 991.36 and 963.15. Hydrochloric acid 4 N and petroleum ether of analytical purity (puriss p.a.) bp 40–60°C (≥90%) were purchased from Sigma‐Aldrich, anti‐bumping granules provided by BDH Laboratory Supplies, and normal phase filtration filters of 180 mm size provided by Whatman (No.1 or No.54, Filters Fioroni).

Proteins were determined with the Kjeldahl method (Büchi AutoKjeldahl Unit K‐370), and their determination was based on the ISO 937‐1978 (for meat and meat products), AOAC Official Methods 920.87‐2010 (partially modified for cereals products) and 991.20‐2011 (for milk and milk products). Wheat flour (certified material FAPAS T2413) concentrated H_2_SO_4_ >95% (for digestion) by Sigma‐Aldrich, CuSO_4_.5H_2_O 99% purity by Scharlau Chemie, K_2_SO_4_ >98% purity (catalysts) and H_2_SO_4_ 0.1 N (for titration) by Merck, NaOH 30%, Η_3_ΒΟ_3_ 2%, and ammonium sulfate (BioUltra ≥ 99.5%) by Sigma‐Aldrich, Na_2_CO_3_.10 H_2_O (900 g/L) by Himedia, sucrose by BDH, and (NH_4_)H_2_PO_4_ purity >99% by Fluka.

Dietary fibers were determined by the enzymatic method, based on AACC 32‐05.01 and AOAC 985.29. Enzymes a‐amylase, protease, and amyloglucosidase were purchased from Megazyme (total Dietary Fibre kit), ethanol 78% and ethanol 95% were provided by Merck, acetone ≥99% and celite provided by Sigma‐Aldrich (acid washed, for total dietary fiber only), NaOH 0.275 Ν (pH 7.5 ± 0.1) provided by Merck (1N), HCl 0.325 N provided by CPA Chem (1 mol/L), and phosphate buffer 0.08 mol/L pH = 6. Certified reference materials (CRMs) FAPAS T2438 and FAPAS T2442 flours and Hanna pH‐meter (Hanna instruments) were also used.

The determination of sugars was partially based on the official AOAC 977.20 method. The separation of sugars was performed by isocratic elution (mobile phase CH_3_CN:H_2_O ‐ 82:18 v/v, column: Water Spherisorb NH_2_ 250 mm × 4.6 mm × 10 μm) with HPLC (HPLC – Alliance, Water 2695, using MILLENIUM software (empowered 3)) and Refractive Index Detector. Sucrose, bioxtra ≥ 99.5%, D (+) glucose 99%, D (+) maltose monohydrate, BioUltra ≥ 99.0%, and α‐Lactose monohydrate were purchased from Sigma‐Aldrich, and D (−) fructose, purity ≥ 99.0% provided by Merck, Carrez I and Carrez II solutions were prepared in‐house (for Carrez I: 219.5 g of zinc acetate (Sigma‐Aldrich, ≥98%) was added to a 100 ml volumetric flask, mixed with 30 ml of acetic acid (Scharlau, D = 1.05 g/cm^3^), and filled with water to the mark, and for Carrez II: 106.0 g of hexacyanoferrate II (BDH 98%) was dissolved in 1,000 ml of water), and Sartorius Stedim membrane filters 0.5 μm were employed.

Determination of minerals was based on AOAC 985.01 and AOAC 984.27. The minerals K, Na, Ca, Mg, P, Cu, Fe, Mn, and Zn were determined by microwave digestion (microwave Ethos 1) in autoclave containers followed by measurement after appropriate dilutions using an Inductively Coupled Plasma with optical emission spectrometer (ICP‐OES, Thermo Scientific, iCAP 6000 Series). HNO_3_ 65% w/w suitable for ICP analysis, H_2_O_2_ 30% w/w with high analytical purity from Merck KGaA, and CRMs were purchased from Merck, standard solutions of minerals, concentrated nitric acid ≥ 69% w/w and 2% v/v solution of HNO_3_ by Carlo erba and the CRM 8435 (whole milk powder) were provided by the National Institute of Standard and Technology.

Determination of caffeine and theobromine was carried out with HPLC ((HPLC – Alliance, Water 2695) and PDA detector (*λ* = 273 nm). The selected mobile phase was MeOH/H_2_O 30:70 v/v, Waters Spherical C_18_ 300 mm × 3.9 mm × 5 μm column was chosen, and the results were processed using Millennium software (empowered 3). Caffeine ≥99% and theobromine ≥99% were purchased from Sigma‐Aldrich, Carrez I and Carrez II solutions were in‐house prepared, and Sartorius Stedim membrane filters 0.5 μm were used. The method limit of detection (LOD) was estimated at 0.085 mg/100 ml for caffeine and 0.265 mg/100 ml for theobromine.

Energy value was calculated based on the energy calculation factors, and the total carbohydrates were calculated by difference.

### 2.2 Data analysis

The results of all the applied analytical methods were statistically proceeded using SIMCA 13.0 (Umetrics, Sweden) for the differentiation of the samples. Each sample was considered as an assembly of 21 variables represented by the chemical data. All the available results were used (Dataset 21 variables × 46 observations × 4 groups, shown in Supporting information Table S1), including all the samples in duplicate (46 samples). Pattern recognition tools used in this work are as follows:

#### 2.2.1 Principal component analysis (PCA)

This procedure was applied mainly to achieve a reduction of dimensionality to permit a primary estimation of the variation in the data matrix (Kokkinofta & Theocharis, 2005). The data were mean‐centered with Unit Variate Scaling (UV), and the PCA model was extracted at a confidence level of 95%.

Partial least square‐discriminant analysis (PLS‐DA): This technique builds classification model by separating the systematic variation in *X* into two parts, one that is linearly related to *Y* (predictive information) and one that is unrelated to *Y*, at a confidence level of 95%. The resulting loading and contribution plots reveal the most discriminating variables (Kokkinofta et al., 2017). The efficiency of the models was described by the goodness‐of‐fit *R* ^2^ (0 ≤ *R* ^2 ^≤ 1) and the predictive ability *Q* ^2^ (0 ≤ *Q* ^2 ^≤ 1) values. The *R* ^2^ explains the variation (how well the data of the training set is mathematically reproduced), while the *Q* ^2^ explains the predictive ability of the model (it represents the fraction of the variation of *Y* that can be predicted). The models have been validated using cross validation‐analysis of variance (CV‐ANOVA), with a *p*‐value < 0.05. For validation purposes, the misclassification table was calculated.

### 2.3 Uncertainty

The results of moisture, ash, and fat were given with their uncertainty (the experiments were held with triplicate samples). Proteins, dietary fibers, and sugars were analyzed in duplicate samples. The carbohydrates uncertainty was found by counting the errors of all methods.

The result of a measurement may deviate from the actual value due to systematic and random errors. Τhe uncertainty was calculated according to EURACHEM/CITAC Guide, 2000 and NORDTEST, 2012.

## 3 RESULTS AND DISCUSSION

### 3.1 Nutritional analysis

**Figure 1:**
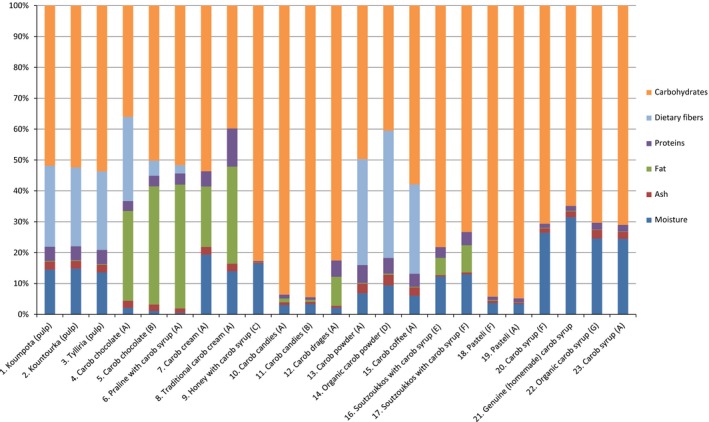
Chemical composition of carobs and carob products

In Figure 1, the results of carbohydrates, dietary fibers, proteins, fat, ash, and moisture are shown. Moisture in carob pods ranges between 13.59% and 14.80% and is in agreement with the literature data (Batlle & Tous, 1997), whereas moisture in carob products falls within 0.46% and 31.44%. The latter values are shown in Table 2, where all the measured parameters are presented in detail and compared with the literature data (Supporting information Table S2). Ash was determined 2.46%–2.63% for carob pods, as also showed by other researchers (Oziyci et al., 2014; Sigge, Lipumbu, & Britz, 2011), and 0.32%–3.24% for carob products. Pods consisted of a very low‐fat content (0.21%–0.23%), as reported also in the literature (Oziyci et al., 2014); however, the percentage of fat is higher in carob products (0.11%–40.13%) because of additional ingredients. Thus, some products end up with a high percentage of fat; this is the case with praline with carob syrup, which is of 40.13% fat. Carob pods along with 12 out of the 20 carob products are low in fat (as they contain less than 3 grams of fat per 100 grams) (Health and nutrition claims, EC Regulation 1924/2006). Carob pods contain 4.54%–4.60% protein, and in most products, the percentage of proteins ranges from 0.43 to 5.12%. However, neither carobs nor their products are sources of protein, as a product is considered source of protein when at least 12% of its energy value is provided by protein (Health and nutrition claims, EC Regulation 1924/2006).

**Table 2:** Results obtained from the analyses of three carob pulps and twenty traditional Cypriot products

Products	Moisture (%) ± U (*n* = 3)	Ash (%) ± U (*n* = 3)	Fat (%) ± U (*n* = 3)	Proteins (%) (*n* = 2)	Dietary fibers (%) (*n* = 2)^a^	Sugars (%) (*n* = 2)	Carbohydrates (%)	Caffeine and theobromine (mg/100 ml) b	Energy value (kcal/100 g)
Koumpota (pulp)	14.48 ± 0.07	2.63 ± 0.07	0.22 ± 0.01	4.57	26.24	53.59	51.86 ± 9.01	<LOD	280.17
Kountourka (pulp)	14.80 ± 0.09	2.49 ± 0.05	0.21 ± 0.02	4.54	25.56	54.03	52.39 ± 9.10	<LOD	280.78
Tylliria (pulp)	13.59 ± 0.11	2.46 ± 0.03	0.23 ± 0.01	4.60	25.43	54.83	53.69 ± 9.33	<LOD	286.07
Carob chocolate A^c^	2.17 ± 0.09	2.39 ± 0.16	29.98 ± 0.45	3.27	28.12	23.15	34.07 ± 5.92	<LOD	475.42
Carob chocolate B	1.09 ± 0.04	2.10 ± 0.02	38.29 ± 0.04	3.41	4.90	56.46	50.21 ± 8.72	<LOD	568.89
Praline with carob syrup A	0.46 ± 0.10	1.45 ± 0.06	40.13 ± 0.06	3.60	2.71	47.83	51.65 ± 8.97	2.51	587.59
Carob cream A	15.50 ± 0.01	2.01 ± 0.02	15.71 ± 0.08	3.95	–	43.04	62.83 ± 10.91	<LOD	408.51
Traditional carob cream A	12.37 ± 0.24	2.15 ± 0.04	27.88 ± 0.08	10.94	–	35.23	46.66 ± 8.11	<LOD	481.32
Honey with carob syrup C	15.84 ± 0.08	0.32 ± 0.05	0.12 ± 0.01	0.43	–	80.07	83.29 ± 14.47	<LOD	335.96
Carob candies A	1.98 ± 0.10	0.57 ± 0.03	0.81 ± 0.07	0.83	–	61.25	95.81 ± 16.64	<LOD	393.85
Carob candies B	2.16 ± 0.05	0.47 ± 0.03	0.42 ± 0.07	0.54	–	61.30	96.41 ± 16.75	<LOD	391.58
Carob drages A	2.05 ± 0.09	0.64 ± 0.03	9.16 ± 0.12	5.11	–	80.32	83.04 ± 14.42	<LOD	435.04
Carob powder A	6.05 ± 0.06	2.74 ± 0.02	0.23 ± 0.02	5.12	30.35	44.01	55.51 ± 9.64	<LOD	305.29
Organic carob powder D	8.49 ± 0.45	3.15 ± 0.06	0.32 ± 0.02	4.58	37.32	36.70	46.14 ± 8.01	<LOD	280.40
Carob coffee A	7.21 ± 0.31	3.24 ± 0.05	0.27 ± 0.01	5.02	34.62	42.32	49.64 ± 8.62	<LOD	290.31
Soutzoukkos with carob syrup E	12.22 ± 0.10	0.49 ± 0.05	5.59 ± 0.27	3.48	–	63.54	78.22 ± 13.59	<LOD	377.11
Soutzoukkos with carob syrup F	12.95 ± 0.08	0.62 ± 0.04	8.83 ± 0.13	4.28	–	52.58	73.32 ± 12.74	<LOD	389.87
Pasteli F	3.66 ± 0.58	0.76 ± 0.09	0.24 ± 0.03	1.07	–	53.61	94.27 ± 16.38	<LOD	383.52
Pasteli A	3.33 ± 0.86	0.44 ± 0.08	0.11 ± 0.01	1.32	–	48.11	94.80 ± 16.47	<LOD	385.47
Carob syrup F	26.43 ± 0.15	1.41 ± 0.06	0.18 ± 0.03	1.35	–	62.14	70.64 ± 12.27	<LOD	289.58
Genuine carob syrup	31.44 ± 0.01	1.91 ± 0.28	0.21 ± 0.09	1.54	–	56.10	64.91 ± 11.27	<LOD	267.60
Organic carob syrup G	24.58 ± 0.08	2.77 ± 0.26	0.13 ± 0.01	2.19	–	55.28	70.33 ± 12.22	<LOD	291.25
Carob syrup A	24.46 ± 0.04	2.29 ± 0.05	0.12 ± 0.01	2.11	–	54.95	71.02 ± 12.34	<LOD	293.60

^a^The analysis for dietary fibers determination was restricted to products marked with dietary fiber, ^b^The LOD for caffeine was estimated 0.085 mg/100 ml and for theobromine 0.265 mg/100 ml, ^c^Where A, B, C, D, E, F, G = different producer

On the other hand, carob chocolate B was found to satisfy the “source of dietary fiber” claim, while carobs and almost all the rest of the analyzed products were found to have a high dietary fiber content, according to Regulation (EU) No. 1924/2006. These products include the following: Cypriot cultivars of carobs (pod), organic carob powder, carob coffee, carob powder, and carob chocolate A. However, praline with carob syrup is not a source of dietary fibers, as it contains less than 3 g of edible fiber per 100 g (Health and nutrition claims, EC Regulation 1924/2006).

Based on EC Regulations No. 1924/2006 and 1169/2011, carobs and their examined products were classified based on the daily reference intakes as a source or a high content in Ca, K, Mg, Na, P, Cu, Fe, Mn, and Zn (Regulation (EU) No 1169/2011). According to Table 3, carob pods are a source of Ca, Cu, Mn, and present a high content of K. Carob syrup contains a high content in K, and the carob powders are a source of Mg, Cu, Fe, and Mn and have a high content in Ca and K. Carob chocolate A and carob chocolate B are a source and/or have a high content in Ca, K, Cu, Fe, Mn, and Zn. The carob cream and the traditional carob cream are sources and/or have a high content in Ca, K, Cu, Fe, Mn, and Zn, and praline with carob syrup is a source of K, P, Fe and has a high content of Cu, Mn. Carob drages are a source of K, Mg, P, Mn and have a high content in Cu. The remaining products (candies, pasteli, soutzoukkos, honey with carob syrup) are neither a source nor have a high content in minerals, as they contain all nine minerals in quantities less than 15% and 30%, respectively, of their daily reference intakes (Regulation (EU) No 1169/2011).

**Table 3:** Minerals of the examined carobs and carob products

Products	Minerals (mg/100 g) (*n* = 2)								
	Ca	K	Mg	Na	P	Cu	Fe	Mn	Zn
Koumpota (pulp)	215	921	50	2.5	65	0.26	0.65	0.38	0.68
Kountourka (pulp)	295	870	44	3.2	57	0.20	0.63	0.44	0.74
Tyllirias (pulp)	204	919	47	1.8	60	0.28	0.66	0.36	0.79
Carob chocolate A	335	677	55	7.1	65	0.56	9.60	0.49	5.80
Carob chocolate B	155	660	35	133	129	0.27	0.56	0.10	0.50
Praline with carob syrup A	96.6	455	47.7	583	137	0.44	3.56	0.61	0.48
Carob cream A	143	835	43	39	129	0.19	9.80	0.28	0.68
Traditional carob cream A	138	312	159	23	305	0.77	4.20	0.97	2.90
Honey with carob syrup C	18	102	6.1	2.1	8.5	<0.01	0.47	0.05	1.40
Carob candies A	18	158	16	5.2	11.0	0.28	1.50	0.07	3.40
Carob candies B	15	120	13	4.7	9	0.14	0.46	0.05	0.49
Carob drages A	86.6	312	63.2	595	114	0.66	1.13	0.51	0.68
Carob powder A	275	968	59	3.4	62	0.32	2.90	0.50	2.10
Organic carob powder D	423	996	62	9.7	63	0.35	5.60	0.55	1.00
Carob coffee A	340	1035	64	5.2	67	0.28	3.90	0.66	0.54
Soutzoukkos with carob syrup E	35	188	35	5.9	67	0.28	0.94	0.55	0.69
Soutzoukkos with carob syrup F	44	238	46	17	91	0.11	0.80	0.77	0.70
Pasteli F	24	218	15	9	17	0.02	0.27	0.12	0.18
Pasteli A	22	210	13.9	10	24	0.04	0.43	0.20	0.30
Carob syrup F	47	628	34	27	45	<0.01	0.50	0.21	2.00
Genuine carob syrup	86	844	71	8.9	41	<0.01	1.80	0.19	3.80
Organic carob syrup G	104	1049	56	28	65	0.06	3.20	0.36	0.45
Carob syrup A	83	1002	52	21	73	0.03	1.30	0.27	0.71
Daily reference intakes									
Daily reference intakes (mg/100 g)	800	2000	375	–	700	1	14	2	10
15% daily reference intake—source (mg/100 g)	120	300	56.25	–	105	0.15	2.1	0.3	1.5
30% daily reference intake—high content (mg/100 g)	240	600	112.5	–	210	0.3	4.2	0.6	3
Literature for carob pods									
(Sigge et al., 2011)	135.67–302.67	852.33–1091.33	55.00–99.00	4.41–14.45	44.00–92.33	0.07–0.23	0.47–0.98	0.59–1.23	0.11–0.69
(Oziyci et al., 2014)	300	970	60	–	71	0.85	1.88	1.29	–
(Khlifa et al., 2013)	266.6–327	993.6–1042	82.75–103	8.47–12.78	68.2–79.7	0.29–0.03	1.78–2.26	0.23–0.30	0.41–0.52

Cypriot carobs are distinguished from other varieties of carobs due to their high sugar content (sucrose, fructose, and glucose). In all three Cypriot cultivars, the measured sugar content was 53.59%–54.83%. These values agree with that of Sigge et al. (Sigge et al., 2011). In carob products, sugars were identified in the range of 23.15% (carob chocolate A) to 80.32% (carob drages). Carbohydrates range from 51.86 to 53.69% in carob pods and 34.07%–96.41% in their products.

Caffeine and theobromine content in carobs pulp, carob powders, and carob coffee were found below the LOD of the method. This is in agreement with the results obtained by other researches (Craig & Nguyen, 1984; Kumazawa et al., 2002). Taking into consideration, the results and the European Food Safety Authority (EFSA) regulation on caffeine levels that energy drinks (except coffee, tea, and cocoa) with caffeine more than 150 mg/L have compulsory “high caffeine content” labeling (“EFSA Panel on Dietetic Products, Nutrition and Allergies,” 2015), carobs and their products are considered caffeine‐free. This corroborates the potential use of carob as an alternative and healthy choice and a coffee and cocoa substitute. Moreover, it has advantages over chocolate, as it has fewer calories and does not contain caffeine or theobromine (Goulas et al., 2016; Khlifa, Bahloul, & Kitane, 2013).

The energy value ranges from 280.17 to 286.07 kcal/100 g in carob pods and from 280.40 to 587.59 kcal/100 g in carob products. Moreover, the results were compared with the nutritional value on product labels (Supporting information Table S3). The labels of eight products made no reference to their nutritional value and simply included their ingredients. This is a known and expectable problem in traditional products due to limited personnel and resources involved. In the absence of research and development departments, products are frequently produced empirically. The examined products derived from small, family enterprises with limited ability to perform frequent nutritional composition analyses. However, after the transitional period of the Regulation (EU No 1169/2011), the nutritional labeling will be mandatory.

It should be stressed that the composition and nutritional quality of carobs are affected mainly by the genotype, the harvest period, the growing conditions, the climatologically conditions (e.g., sun, and water availability), the soil content, and the overall microclimate. In the same way, the postharvest conditions (e.g., storage and distribution of the food chain) should not be underestimated.

### 3.2 Chemometric analysis

First, basic statistics was performed in order to extract the correlation between the original variables (nutritional components). The correlation matrix is presented in Supporting information Table S4 a. Proteins, Mg, P, and Mn are highly correlated, while Cu has a smaller but significant correlation with proteins and P. Finally, glucose shows good correlation with Ca and P. Boxplots in Supporting information Figure S1 displays the components in vertical plots and provides a visual comparison of the spread of the distributions, helping to detect outliers among the different type of carob products.

**Figure 2:**
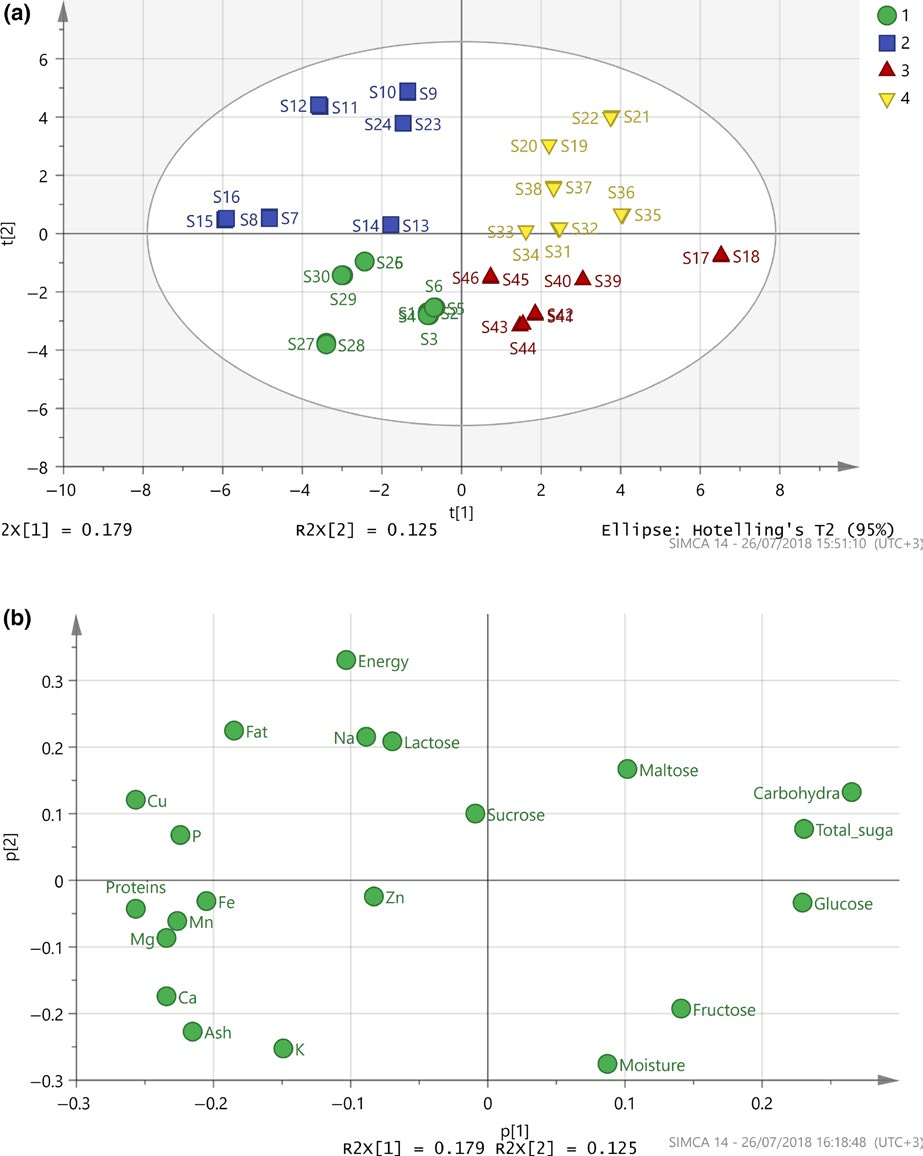
(a) Score plot of PCA model, *N* = 46, *R* ^2^(cum) = 0.89, *Q* ^2^(cum) = 0.54 (1: circle = powder/coffee, 2: rectangular = creamy product, 3: triangle = carob syrup, 4: inverted triangle = pasteli/soutzoukkos). (b) Loading plot

Principal component analysis was performed on the 46 samples in four preformed groups (powder/coffee, creamy products, carob syrup, and pasteli/soutzoukkos), to estimate the systematic variation in a data matrix by a low‐dimensional model plane. After scaling, to eliminate the effect of different size variables, the obtained results are shown in Figure 2. With regard to the overall PCA, the first two components explain the 54.9% of the data variation, with predictability *Q* ^2^ (cum) = 0.549. The four groups are well separated in the cloud of points, although the group of the creamy products of carob showed wide variation. The main differentiation depicted in the model along the first principle component reflects their different composition, which depends on the raw material that is affected by the specific geoclimatic conditions existing in the production area of carobs (Figure 2a). The components responsible for this differentiation are the carbohydrates, which characterize the group of the traditional pasteli and soutzoukko. The group of carob syrup differentiates mainly due to its glucose content, which is significantly higher in the products from mixing carob syrup and honey (S17, S18).

Furthermore, along the second principle component, a metal‐based trend was observed, as shown in the loading plot (Figure 2b). The creamy products of carob are strongly characterized by sodium, while calcium is responsible for distinguishing between the samples of pulp, powder, and coffee, which appear very close with great similarity.

**Figure 3:**
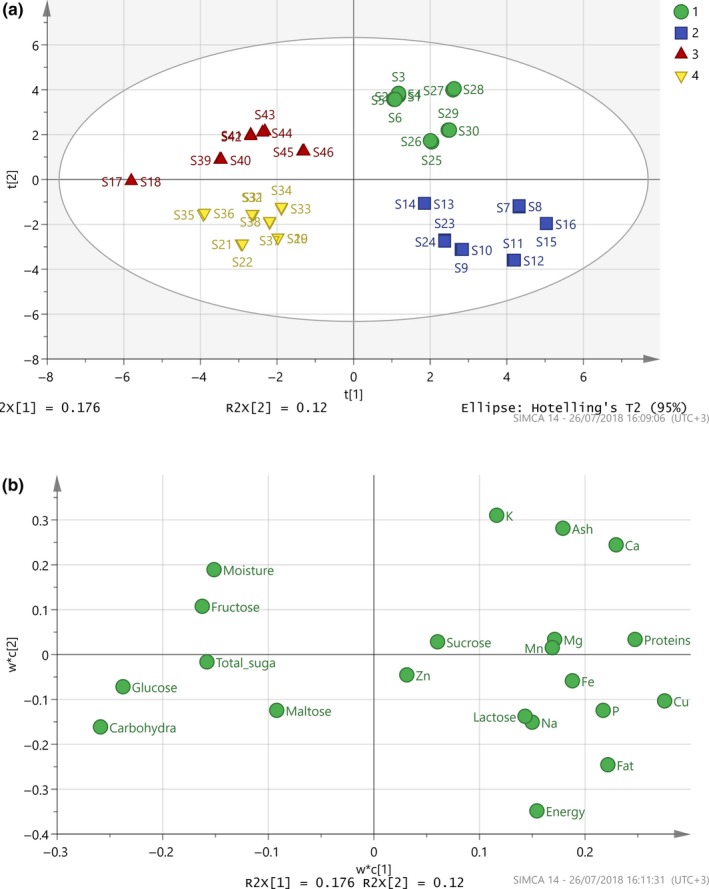
(a) Score plot of PLS‐DA model, *N* = 46, *R* ^2^(cum) = 0.82, *Q* ^2^(cum) = 0.90 (1: circle = powder/coffee, 2: rectangular = creamy product, 3: triangle = carob syrup, 4: inverted triangle = pasteli/soutzoukkos). (b) Loading plot

Partial least square‐discriminant analysis was applied to validate the previous results on the influence of the production process to the composition. The discrimination was satisfactorily correct, although the above mixtures of carob syrup and honey classified as “pasteli” that is not entirely wrong, considering similar production (Figure 3). The extracted PLS‐DA model depicted very high values in terms of its sensitivity (*R* ^2^ = 0.82, *Q* ^2^ = 0.90). The misclassification matrix in Supporting information Table S4 b calculated without and with one‐out‐cross validation shows how well the classes are separated. The rows correspond to the true classes; the columns correspond to the assigned classes. Diagonal values are number of correct classification. The fishers’ probability is highly satisfactory (2.4^10^−25^ < 0.05), and these findings confirm the earlier conclusions from the PCA model.

## 4 CONCLUSIONS

A holistic approach was followed for the nutritional analysis of carobs, from the fruit to the final commercial product. The chemical composition of Cyprus carob cultivars was examined for the first time, and the results were compared with that of commercially available carob‐based traditional products using chemometrics tools. The possibility of characterized carobs as functional foods (fat, dietary fibers, and minerals) was explored, taking into consideration the presence of many antioxidant components (e.g., polyphenols and flavanols), as indicated in the previous literature studies (Stavrou, Christou, & Kapnissi‐Christodoulou, 2018). Additionally, carobs contain high amounts of D‐pinitol (3‐O‐methyl‐chiroinositol), a natural bioactive and effective ingredient, with proven insulin‐like function (Bates, Jones, & Bailey, 2000). According to the current results, the nutritional or chemical composition of foods changes if the amount and type of ingredients used in the formulation differed. The examined food products are usually commercially available in small local markets, and traditional products suffer from standardization, internal national control on product identity, and the requirement for strong nutritional labeling and/or packaging.

Modern diet and lifestyle are associated with severe diseases in the Southern and Eastern Mediterranean (e.g., obesity, diabetes, and cardiovascular disease), and therefore, biological food market is growing. Carobs appear to fulfill modern health criteria of consumers (i.e., gluten‐ and caffeine‐free product, natural chocolate‐like sweetener, ingredient for bread, beverages such as liqueurs and tea, nuts, tahini, and honey). Local efforts for PDO and PGI products can be supported and enhanced within Europe, especially when such fruits and products are cultivated and linked with the tradition and history of many European countries (e.g., Spain, Italy, Greece, Portugal, and Cyprus). Besides, in the old days, the consumption of fruits, nuts, and cereals was part of the Mediterranean diet.

Therefore, carob and carob powders are proposed to be included in humans’ daily diet as they contain valuable nutrients, low fat and have a sweet taste. However, this is not the case for all carob products, as some of them were determined with a high‐fat content, and they are neither a source of edible fibers nor minerals. Carob products partially satisfied health and nutritional claims: 60% in terms of fat, 25% in terms of dietary fibers, and 80% in terms of for minerals. Moreover, although containing enough percentage of fat, some products also contain enough nutrients. Given the nutritional value of carob, the indication that commercial products contain carob may be misleading for some consumers because they buy these products thinking they are functional foods. Nevertheless, depending on the manufacturing processing, carobs could be used as a natural ingredient for the creation of new functional foods based on the high fiber and minerals content and low fat levels. Carob, indigenous in Cyprus, is of high biological value and must be protected. Standardization of carob products is proposed in order to (a) ensure proper consumer information and protection against misleading indications in product labeling and (b) to protect producers from unfair practices in the production of carob products.

In summary, this research assesses for the first time the nutritional components in different carob products, in order to provide tools for their characterization as functional food products. This study can be considered as a pilot; more samples could be analyzed to enhance the above conclusions. Nevertheless, it contributes to nutritional estimation of carob and gives added value to Cypriot traditional carob products.

## CONFLICT OF INTEREST

The authors declare that there is no conflict of interest.

## ETHICAL STATEMENT

This study does not involve any human or animal testing.
