# Supplementary material for: Nutritional characterization of carobs and traditional carob products
Source: Food Sci Nutr. 2018 Oct 4;6(8):2151–61. doi: 10.1002/fsn3.776 (PMC6261171; doi:10.1002/fsn3.776)
Supplement: Supplementary file 1 [file FSN3-6-2151-s001.docx]

**Supplementary Material**

**Nutritional characterization of carobs and traditional carob products**

Eleni Papaefstathiou^1^, Agapios Agapiou^1*^, Stelios Giannopoulos^2^, Rebecca Kokkinofta^2^

*^1^University of Cyprus, Department of Chemistry, P.O. Box 20537, 1678, Nicosia, Cyprus*

*^2^State General Laboratory, Nicosia, Cyprus*

**Corresponding author: Tel.: +357-22-895432; fax: +357-22-895466*

*E-mail address:* [agapiou.agapios@ucy.ac.cy](mailto:agapiou.agapios@ucy.ac.cy)

**Table SM1.** Dataset of measurements used for Chemometric analyses.

| **Observations** | **Group** | **Type** |
| --- | --- | --- |
| S1, S2 | 1 | Koumpota (pulp) |
| S3, S4 | 1 | Kountourka (pulp) |
| S5, S6 | 1 | Tylliria (pulp) |
| S7, S8 | 2 | Carob chocolate A |
| S9, S10 | 2 | Carob chocolate B |
| S11, S12 | 2 | Praline with carob syrup A |
| S13, S14 | 2 | Carob cream A |
| S15, S16 | 2 | Traditional carob cream A |
| S17, S18 | 3 | Honey with carob syrup C |
| S19, S20 | 4 | Carob candies A |
| S21, S22 | 4 | Carob candies B |
| S23, S24 | 2 | Carob drags A |
| S25, S26 | 1 | Carob powder A |
| S27, S28 | 1 | Organic carob powder D |
| S29, S30 | 1 | Carob coffee A |
| S31, S32 | 4 | Soutzoukkos with carob syrup E |
| S33, S34 | 4 | Soutzoukkos with carob syrup F |
| S35, S36 | 4 | Pasteli F |
| S37, S38 | 4 | Pasteli A |
| S39, S40 | 3 | Carob syrup F |
| S41, S42 | 3 | Genuine carob syrup |
| S43, S44 | 3 | Organic carob syrup G |
| S45, S46 | 3 | Carob syrup A |

**Table SM2.** Chemical composition (%) of carobs according to literature.

| **Carob Pod** | **Chemical composition (%)** |
| --- | --- |
|  | **(according to literature)** |
| Moisture | 8-10 (Sigge et al., 2011)  10-20 (Batlle & Tous, 1997) |
| Total sugars | 40-55 (Haber, 2002), (Goulas et al., 2016)  40.69-54.74 (Sigge et al., 2011) |
| Sucrose | 32-38 (Batlle & Tous, 1997)  29.8-63.5 (Biner, Gubbuk, Karhan, Aksu, & Pekmezci, 2007) |
| Glucose | 5-6 (Batlle & Tous, 1997)  1.8-10.2 (Goulas et al., 2016) |
| Fructose | 5-7 (Batlle & Tous, 1997)  1.8-12.5 (Goulas et al., 2016) |
| Dietary Fibers | 30-40 (Haber, 2002)  11 (Oziyci et al., 2014) |
| Proteins | 1.00-5.00 (Oziyci et al., 2014)  3.07-4.42 (between 1-7.6) (Sigge et al., 2011) |
| Fat^,^ | 0.45-0.86 (Sigge et al., 2011)  0.2-0.8 (Oziyci et al., 2014) |
| Ash | 2.13-2.69 (Sigge et al., 2011)  2.04-4.37^,^(Oziyci et al., 2014) |

**Table SM3.** Comparison between the results and the nutritional value on product labels.

| **Products** | **Label** | **Results** |
| --- | --- | --- |
| Carob chocolate A | Moisture: 2,22 %  Ash: 1,83 %  Fat: 20,8 %  Proteins: 3,8 %  Dietary Fibers: 11,4 %  Carbohydrates: 71,4 %  Sugars: 26,9 %  Energy: 442 kcal/100g | Moisture: 2,17  Ash: 2,39 %  Fat: 29,98 %  Proteins: 3,27 %  Dietary Fibers: 28,12 %  Sugars: 23,15 %  Carbohydrates: 34,07 %  Energy: 475,42 kcal/100g |
| Carob chocolate B | - | Moisture: 1,09 %  Ash: 2,10 %  Fat: 38,29 %  Proteins: 3,41 %  Dietary Fibers: 4,90 %  Sugars: 56,46 %  Carbohydrates: 50,21 %  Energy: 568,89 kcal/100g |
| Praline with carob syrup A | Fat: 33,5 %  Proteins: 4,8 %  Dietary Fibers: 1,1 %  Sugars: 55,4 %  Carbohydrates: 58,7 %  Energy: 558 kcal/100g | Moisture: 0,46 %  Ash: 1,45 %  Fat: 40,13 %  Proteins: 3,60 %  Dietary Fibers: 2,71 %  Sugars: 47,83 %  Carbohydrates: 51,65 %  Energy: 587,59 kcal/100g |
| Carob cream A | Fat: 15,1 %  Proteins: 3,4 %  Sugars: 42,6 %  Carbohydrates: 63,3 %  Energy: 403 kcal/100g | Moisture: 15,50 %  Ash: 2,01 %  Fat: 15,71 %  Proteins: 3,95 %  Dietary Fibers: -  Sugars: 43,04 %  Carbohydrates: 62,83 %  Energy: 408,51 kcal/100g |
| Traditional carob cream A | Moisture: 47 %  Ash: 3 %  Fat: 27 %  Proteins: 8,5 %  Carbohydrates: 53 %  Energy: 510 kcal/100g | Moisture: 12,37 %  Ash: 2,15 %  Fat: 27,88 %  Proteins: 10,94 %  Dietary Fibers: -  Sugars: 35,23 %  Carbohydrates: 46,66 %  Energy: 481,32 kcal/100g |
| Honey with carob syrup C | Fat: 0 %  Proteins: 1 %  Carbohydrates: 79 %  Energy: 290 kcal/100g | Moisture: 15,84 %  Ash: 0,32 %  Fat: 0,12 %  Proteins: 0,43 %  Dietary Fibers: -  Sugars: 80,07 %  Carbohydrates: 83,29 %  Energy: 335,96 kcal/100g |
| Carob candies A | - | Moisture: 1,98 %  Ash: 0,57 %  Fat: 0,81 %  Proteins: 0,83 %  Dietary Fibers: -  Sugars: 61,25 %  Carbohydrates: 95,81 %  Energy: 393,85 kcal/100g |
| Carob candies B | Moisture: 45,1 %  Fat: 0 %  Proteins: 0,2 %  Dietary Fibers: 0,5 %  Sugars: 45,4 %  Carbohydrates: 97,5 %  Energy: 391 kcal/100g | Moisture: 2,16 %  Ash: 0,47 %  Fat: 0,42 %  Proteins: 0,54 %  Dietary Fibers: -  Sugars: 61,30 %  Carbohydrates: 96,41 %  Energy: 391,58 kcal/100g |
| Carob drages A | - | Moisture: 2,05 %  Ash: 0,64 %  Fat: 9,16 %  Proteins: 5,11 %  Dietary Fibers: -  Sugars: 80,32 %  Carbohydrates: 83,04 %  Energy: 435,04 kcal/100g |
| Carob powder A | Moisture: 6,14 %  Ash: 3,04 %  Fat: 0,2 %  Proteins: 4,6 %  Dietary Fibers: 48,5 %  Sugars: 37,5 %  Carbohydrates: 86 %  Energy: 170 kcal/100g | Moisture: 6,05 %  Ash: 2,74 %  Fat: 0,23 %  Proteins: 5,12 %  Dietary Fibers: 30,35 %  Sugars: 44,01 %  Carbohydrates: 55,51 %  Energy: 305,29 kcal/100g |
| Organic carob powder D | - | Moisture: 8,49 %  Ash: 3,15 %  Fat: 0,32 %  Proteins: 4,58 %  Dietary Fibers: 37,32 %  Sugars: 36,70 %  Carbohydrates: 46,14 %  Energy: 280,40 kcal/100g |
| Carob coffee A | Moisture: 6,14 %  Ash: 3,04 %  Fat: 0,2 %  Proteins: 4,6 %  Dietary Fibers: 48,5 %  Sugars: 37,5 %  Carbohydrates: 86,0 %  Energy: 170 kcal/100g | Moisture: 7,21 %  Ash: 3,24 %  Fat: 0,27 %  Proteins: 5,02 %  Dietary Fibers: 34,62 %  Sugars: 42,32 %  Carbohydrates: 49,64 %  Energy: 290,31 kcal/100g |
| Soutzoukkos with carob syrup E | Fat: 6 %  Proteins: 4,5 %  Sugars: 40 %  Carbohydrates: 66 %  Energy: 338 kcal/100g | Moisture: 12,22 %  Ash: 0,49 %  Fat: 5,59 %  Proteins: 3,48 %  Dietary Fibers: -  Sugars: 63,54 %  Carbohydrates: 78,22 %  Energy: 377,11 kcal/100g |
| Soutzoukkos with carob syrup F | - | Moisture: 12,95 %  Ash: 0,62 %  Fat: 8,83 %  Proteins: 4,28 %  Dietary Fibers: -  Sugars: 52,58 %  Carbohydrates: 73,32 %  Energy: 389,87 kcal/100g |
| Pasteli F | - | Moisture: 3,66 %  Ash: 0,76 %  Fat: 0,24 %  Proteins: 1,07 %  Dietary Fibers: -  Sugars: 53,61 %  Carbohydrates: 94,27 %  Energy: 383,52 kcal/100g |
| Pasteli A | Fat: 0,1 %  Proteins: 0,8 %  Dietary Fibers: 0,4 %  Sugars: 47,8 %  Carbohydrates: 49,8 %  Energy: 204 kcal/100g | Moisture: 3,33 %  Ash: 0,44 %  Fat: 0,11 %  Proteins: 1,32 %  Dietary Fibers: -  Sugars: 48,11 %  Carbohydrates: 94,80 %  Energy: 385,47 kcal/100g |
| Carob Syrup F | - | Moisture: 26,43 %  Ash: 1,41 %  Fat: 0,18 %  Proteins: 1,35 %  Dietary Fibers: -  Sugars: 62,14 %  Carbohydrates: 70,64 %  Energy: 289,58 kcal/100g |
| Genuine carob syrup | - | Moisture: 31,44 %  Ash: 1,91 %  Fat: 0,21 %  Proteins: 1,54 %  Dietary Fibers: -  Sugars: 56,10 %  Carbohydrates: 64,91 %  Energy: 267,60 kcal/100g |
| Organic carob syrup G | Fat: 0,4 %  Proteins: 1,8 %  Carbohydrates: 67,7 %  Energy: 270 kcal/100mL | Moisture: 24,58 %  Ash: 2,77 %  Fat: 0,13 %  Proteins: 2,19 %  Dietary Fibers: -  Sugars: 55,28 %  Carbohydrates: 70,33 %  Energy: 291,25 kcal/100g |
| Carob syrup A | Fat: 0,6 %  Proteins: 1,7 %  Sugars: 48,5 %  Carbohydrates: 63,8 %  Energy: 267,2 kcal/100g | Moisture: 24,46 %  Ash: 2,29 %  Fat: 0,12 %  Proteins: 2,11 %  Dietary Fibers: -  Sugars: 54,95 %  Carbohydrates: 71,02 %  Energy: 293,60 % |

**Table SM4. (a)** Correlation matrix of the original variables (nutritional components).


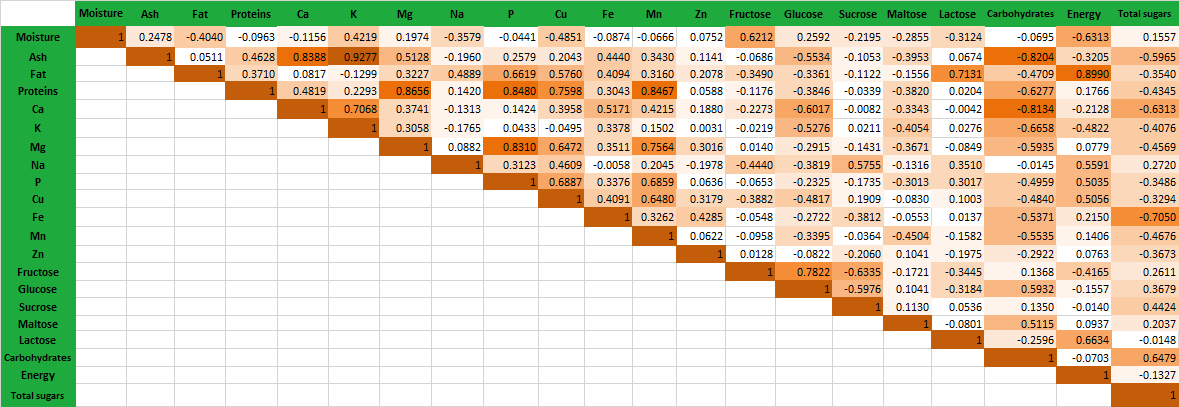


**Table SM4. (b)** PLS-DA classification.

|  | **Members** | **Correct** | **1** | **2** | **3** | **4** | **No class (YPred < 0)** |
| --- | --- | --- | --- | --- | --- | --- | --- |
| **1** | 12 | 100% | 12 | 0 | 0 | 0 | 0 |
| **2** | 12 | 100% | 0 | 12 | 0 | 0 | 0 |
| **3** | 10 | 100% | 0 | 0 | 10 | 0 | 0 |
| **4** | 12 | 100% | 0 | 0 | 0 | 12 | 0 |
| **No class** | 0 |  | 0 | 0 | 0 | 0 | 0 |
| Total | 46 | 100% | 12 | 12 | 10 | 12 | 0 |
| Fishers prob. | 2.4e-025 |  |  |  |  |  |  |

**Figure SM1. (a)** Boxplots of nutritional components of the different types of carob’s products.

| 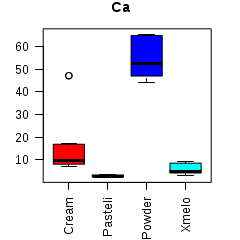  * | 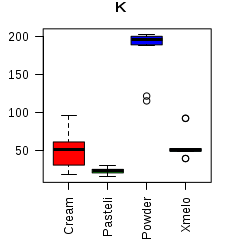 | 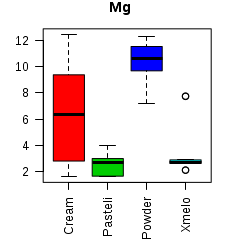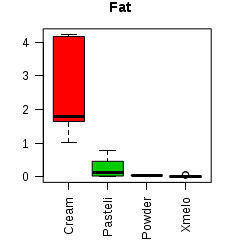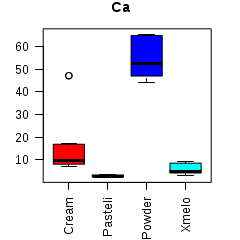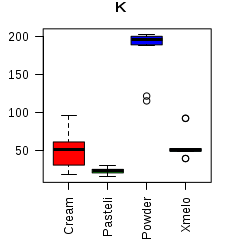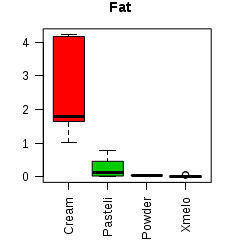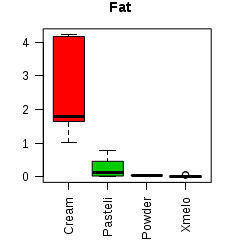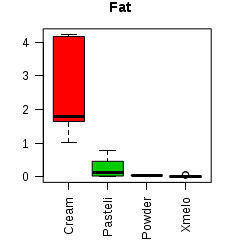 |
| --- | --- | --- |
| 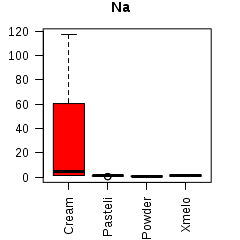 | 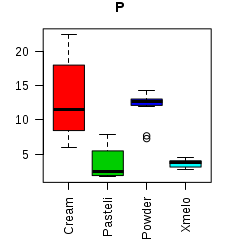 | 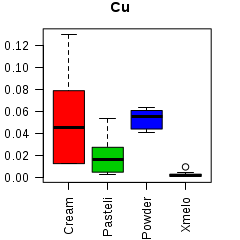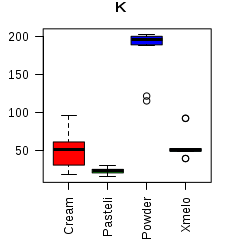 |
| 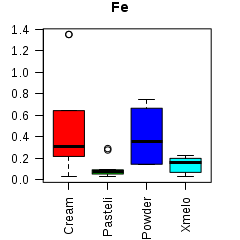 | 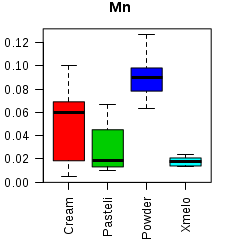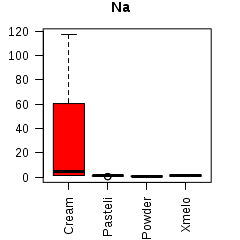 | 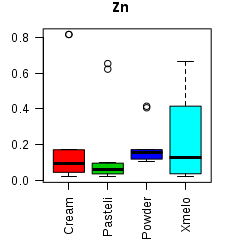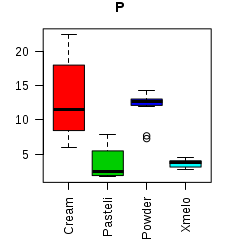 |

*Where Xmelo = carob syrup

**Figure SM1. (b)** Boxplots of nutritional components of the different types of carob’s products.

| 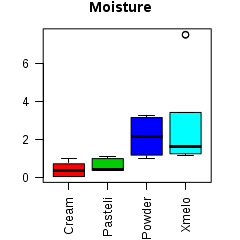 | 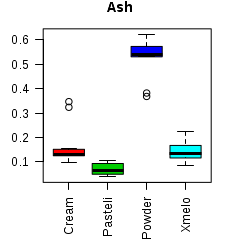 | 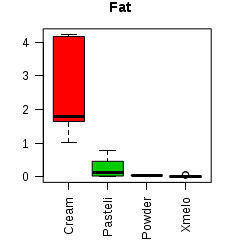 |
| --- | --- | --- |
| 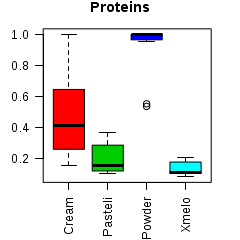 | 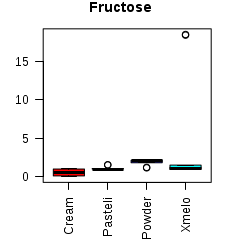 | 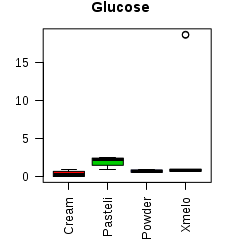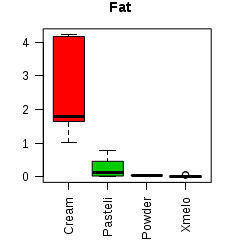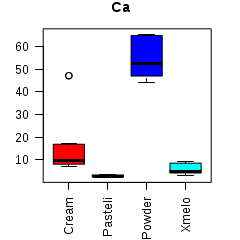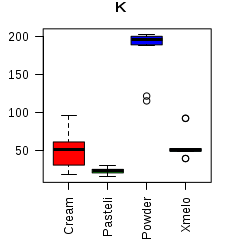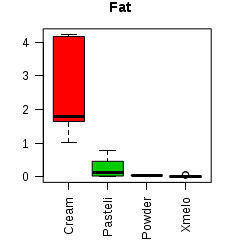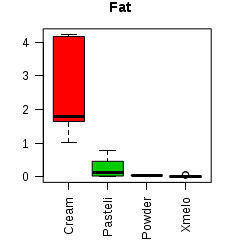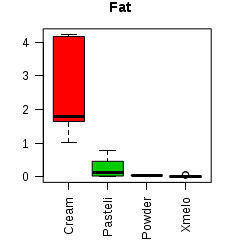 |
| 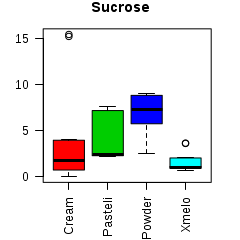 | 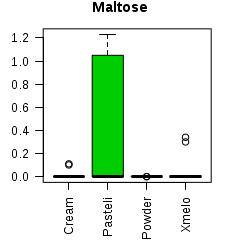 | 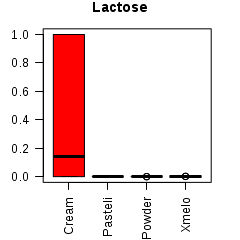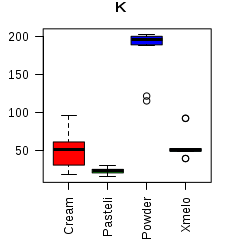 |
| 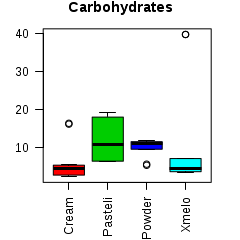 | 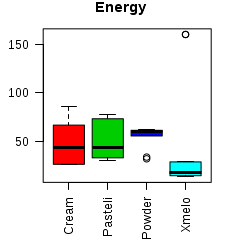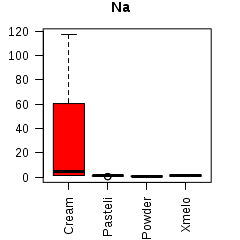 | 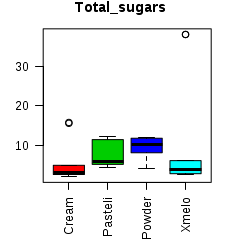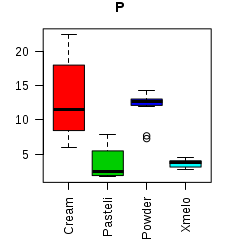 |
